# Accurate prenatal diagnosis of coarctation of the aorta by 3-step echocardiographic diagnostic protocol

**DOI:** 10.1186/s12887-024-04851-7

**Published:** 2024-08-27

**Authors:** Hong Meng, Zhi-Ling Luo, Yan Shen, Qian-Qian Liu, Mu-Zi Li, Yi-Ming Gao

**Affiliations:** 1 Department of Echocardiography, Fuwai Hospital, National Center for Cardiovascular Diseases, Chinese Academy of Medical Sciences and Peking Union Medical College, 167 Beilishi Rd, Xicheng District, Beijing, 100037 P. R. China; 2 Department of Echocardiography, Fuwai Yunnan Cardiovascular Hospital Kunming, Kunming, 650102 China

**Keywords:** Aortic arch, TAO-DAO angle, Coarctation of the aorta, Fetal echocardiography

## Abstract

**Background:**

Coarctation of the aorta (CoA) is the most common undiagnosed congenital heart defect during prenatal screening. High false positive and false negative rates seriously affect prenatal consultation and postnatal management. The objective of the study was to assess the utility of various measurements to predict prenatal CoA and to derive a diagnostic algorithm.

**Methods:**

One hundred and fifty-four fetuses with suspected CoA who presented at Fuwai Hospital between December 2017 and August 2021 were enrolled and divided into confirmed CoA cases (*n* = 47) and false positive cases (*n* = 107), according to their postnatal outcomes. The transverse aortic arch, isthmus, and descending aorta were measured in the long-axis view of the aortic arch. The angle between the transverse aortic arch (TAO) and the descending aortic arch (DAO) was defined as the TAO-DAO angle and measured in the long axis or sagittal view. Based on the database in GE Voluson E10 and the formula (Z = $\frac{\text{x}-{\mu }}{\alpha }$), the standard score (Z-score) of the dimensions of the aorta were calculated in relation to the gestational age. The main echocardiographic indices were combined to design a 3-step diagnostic protocol. The TAO-DAO angle was used as the first step in the diagnostic model. The diameter of the transverse arch and the Z-score of the isthmus were the second step. The third-step indices included a Z-score of the transverse arch, diameter of the isthmus, distance from the left subclavian artery (LSA) to left common carotid artery (LCCA), the ratio of isthmus diameter and LSA diameter and ratio of the distances (the distance between the LSA and LCCA to the distance between the right innominate artery and LCCA). The receiver operating characteristic (ROC) curve determined the predictive capability of each diagnostic parameter, and the kappa test determined the diagnostic accuracy of the proposed model.

**Results:**

The cases with confirmed CoA had thinner transverse arches (1.92 ± 0.32 mm vs. 3.06 ± 0.67 mm, *P* = 0.0001), lower Z-scores of the isthmus (-8.97 ± 1.45 vs. -5.65 ± 1.60, *P* = 0.0001), smaller TAO-DAO angles (105.54 ± 11.51° vs. 125.29 ± 8.97°, *P* = 0.0001) and larger distance between the LSA and LCCA (4.45 ± 1.75 mm vs. 2.74 ± 1.07 mm, *P* = 0.0001) than the false positive cases. The area under the curve (AUC) was 0.947 (95% CI 0.91–0.98) for the TAO-DAO angle ≤ 115.75°, 0.942 (95% CI 0.91–0.98) for the transverse arch diameter ≤ 2.31 mm, 0.937 (95% CI 0.90–0.98) for the Z-score of the isthmus ≤ -7.5, and 0.975 (95% CI 0.95–1.00) for the 3-step diagnostic protocol with 97.8% sensitivity and 97.2% specificity. The kappa test showed that the model’s diagnostic accuracy was consistent with postnatal outcomes (kappa value 0.936, *P* = 0.0001).

**Conclusions:**

The 3-step diagnostic protocol included the three most useful measurements and the additional indices with appropriate cut-off values. The algorithm is useful for the detection of aortic coarctation in fetuses with a high degree of accuracy.

**Trial registration:**

Retrospectively registered.

## Background

Coarctation of the aorta (CoA) is the most common undiagnosed congenital heart defect during prenatal screening, with a true positive detection rate of < 50% [1, 2]. Accurate prenatal diagnosis allows planning of the delivery in a center with a pediatric cardiology service, maintaining arterial duct patency by starting prostaglandin infusion immediately after birth, and performing surgery timely, thus reducing mortality and morbidity. High false positive and false negative rates seriously affect prenatal consultation and postnatal management, causing unnecessary parental anxiety and hospitalization costs. The main limitations to the prenatal diagnosis of CoA are the low specificity of the classic echocardiographic signs (ventricular disproportion, great vessel asymmetry, shelf of the aortic isthmus) and the hard-to-predict arch remodeling after the closure of the patent ductus arteriosus (PDA) [3, 4]. Even though some authors have studied the Z-scores of cardiovascular dimensions to identify fetal CoA [5, 6], it cannot be reliably diagnosed using a single parameter, and clinicians should maintain a high index of suspicion when multiple parameters are outside of the expected ranges.

The objective of this retrospective study was to summarize the diagnostic performance of various echocardiographic measurements for detecting fetal CoA and to derive an optimal diagnostic algorithm.

## Materials and methods

Fetuses presenting with suspected CoA based on right ventricular dominance and a small diameter of the aortic arch or isthmus between December 2017 and August 2021 were included in the study. They underwent fetal echocardiography at Fuwai Hospital and Branch Hospital. The exclusion criteria were prenatally suspected hypoplastic left heart syndrome, complex congenital heart defect (CHD), and insufficient technical quality including suboptimal imaging. The Institutional Review Board of Fuwai Hospital approved the study protocol (FZX2019-06-01 2022YFKY085), which was in accordance with the “Declaration of Helsinki.”

The participants were divided into confirmed or false positive cases according to the postnatal echocardiography or cardiac computed tomography (CT) results. Pregnant women and their families were informed about the limitations of fetal echocardiography before the examination and signed an informed consent form for the performance of the exam. The consent forms to collect data for research were obtained during follow-up after birth. Echocardiography was performed by two experienced doctors (H. M. and L.Z.L.), and all measurements were performed by an experienced doctor (H.M).

Fetal echocardiography was performed on a GE Voluson E10 ultrasound machine (GE Medical Systems, Austria). Measurements were taken according to published standards and included the right ventricular (RV) end diastolic dimension (RVEDD), left ventricular (LV) end diastolic dimension (LVEDD), LV end diastolic area (LVEDA), RV end diastolic area (RVEDA), mitral valve (MV) and tricuspid valve (TV) annular diameters in the 4-chamber view, aortic valve (AV) annular diameter and ascending aorta diameter in the LV outflow view, pulmonary valve (PV) annular diameter and main pulmonary artery diameter in the RV outflow view, aortic artery (AO) and pulmonary artery (PA) diameters in the three vessels and trachea view (3VT).

The transverse aortic arch, isthmus, and descending aorta should be clearly shown in the long axis view of the aortic arch. The diameter of the left subclavian artery (LSA), distance between the LSA and left common carotid artery (LCCA), distance between the right innominate artery (RIA) and LCCA, and diameter of the distal transverse aortic arch (the aortic arch segment between the LCCA and LSA) were measured. The aortic isthmus should either be measured proximal to the insertion of the PDA in the sagittal and coronary views or may be measured proximal to the orifice of the LSA in the long axis view.

**Fig. 1:**
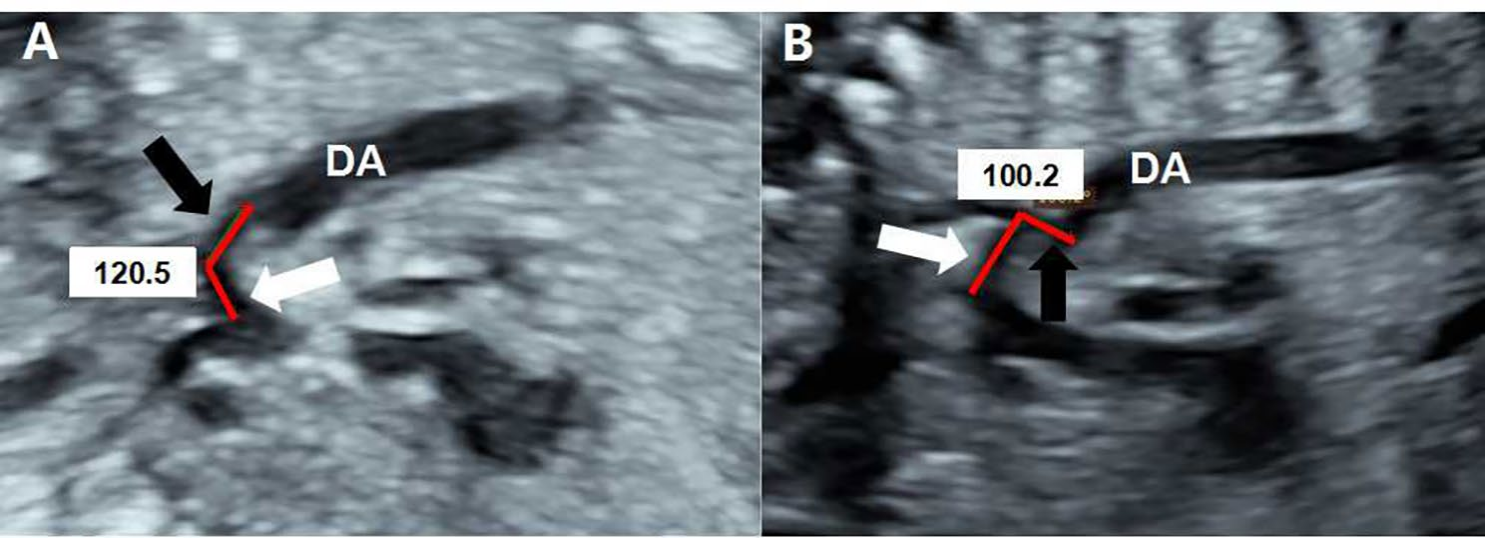
Echocardiographic images depicting the measurement of the TAO-DAO angle. In the long axis or sagittal view, one line is drawn from the origin of the left common carotid artery to the end of the left subclavian artery parallel with the transverse arch (white arrow), and the second line is drawn prior to the isthmus to the end of the isthmus (black arrow). The aortic arch has a smooth TAO-DAO angle of 120.5° in a fetus without coarctation of the aorta (**A**). The aortic arch presents a rigid contour with a sharp TAO-DAO angle of 100.2° in a fetus with coarctation of the aorta (**B**). DA, descending thoracic aorta

The angle between the transverse aortic arch (TAO) and the descending aortic arch (DAO) was defined as the TAO-DAO angle. In the long axis or sagittal view, the angle was measured by two straight lines, where one line was drawn proximal (from the take-off of the second head vessel) to distal (to the end of the third head vessel) along the internal boundary of the transverse arch, and the second line was drawn proximal (just prior to the isthmus) to distal (to the end of the isthmus) along the internal boundary of the isthmus (Fig. 1) [3, 7].

Based on the formula (Z = $\frac{\text{x}-{\mu }}{\alpha }$), the standard score (Z-score) of the valve’s annular diameters and the dimensions of the aorta were calculated in relation to the gestational age [8]. The RVEDD-to-LVEDD ratio, RVEDA-to-LVEDA ratio, MV-to-TV ratio, isthmus-to-LSA ratio, AO-to-PA ratio, and the ratio of the distances (the distance between the LSA and LCCA to the distance between the RIA and LCCA) were calculated. The flow directions across the atrial septum and across the isthmus were observed. Abnormal blood flow across the isthmus were included a retrograde or delayed passage.

After birth, all participants were followed up using echocardiography, and postnatal outcomes were obtained through medical records and parental telephonic follow-up. The diagnosis of CoA after birth was based on (1) the diameters of the proximal and distal transverse aortic arch and aortic isthmus being less than 60%, 50%, and 40%, respectively, of the distal ascending AO; (2) the diameter of the transverse aortic arch being less than 50% of the descending AO at the diaphragm; (3) the diameter of the transverse aortic arch being less than one plus the neonatal weight; (4) the peak coarctation velocity being ≥ 2.5 m/s (> 2.0 m/s for neonates); and (5) a brachial-ankle blood pressure difference > 20 mmHg [9]. However, in neonates with a wide-open ductus arteriosus, it is difficult to evaluate the degree of CoA.

Indications for intervention (surgery or catheterization) were (1) systolic upper- to lower extremity blood pressure gradient > 20 mmHg on non-invasive blood pressure measurement, echocardiography, or cardiac catheterization; (2) diminished or absent lower extremity pulses; (3) systemic hypertension in the presence of significant coarctation; (4) heart failure attributable to coarctation (with increasing severity, the presentation can extend to diminished cardiac output, poor end organ perfusion, and cardiogenic shock); (5) radiologic evidence of significant collateralization; (6) neonates with PGE-dependent CoA [10, 11]. In neonates with these diagnostic clues, initial treatment consisted of PGE1 followed by intervention. If coarctation correction was not required in the neonatal period, the children underwent cardiologic and echocardiographic follow-ups at 3, 6, and 12 months of age. Cardiac CT was performed to confirm the diagnosis before surgery.

The continuous data are expressed as mean ± SD, and categorical data are expressed as frequency and percentage. Continuous variables were analyzed using the t-test, and categorical variables were analyzed using the chi-square test. The receiver operating characteristic (ROC) curve determined the predictive capability of each diagnostic parameter. The cut-off points with the best balance between specificity and sensitivity were established. The kappa test measured the disagreement and agreement between the observers or indices.

## Results

One hundred and fifty-four fetuses with suspected CoA were enrolled in the study. Postnatal echocardiography or cardiac CT confirmed the diagnosis of CoA in 47 cases, and that of normal aortic arches in 107 false positive cases. The mean maternal age was 30.0 ± 4.5 years (range, 21–43 years). The mean gestational age at diagnosis was 28.1 ± 4.1 weeks (range, 20–39 weeks) in 154 fetuses. Fifty-four fetuses (35.1%) had ventricular septal defects (VSD), and 19 (12.3%) had persistent left superior venae cavae (PLSVC). The flow direction across the atrial septum was right to left in all fetuses. Fourteen fetuses with limited atrial shunt (< 3 mm) among 107 false positive cases, while five fetuses among 47 confirmed cases (*P* > 0.05). The characteristic of the flow across the isthmus was different between the two groups (*P* = 0.0001).

**Fig. 2:**
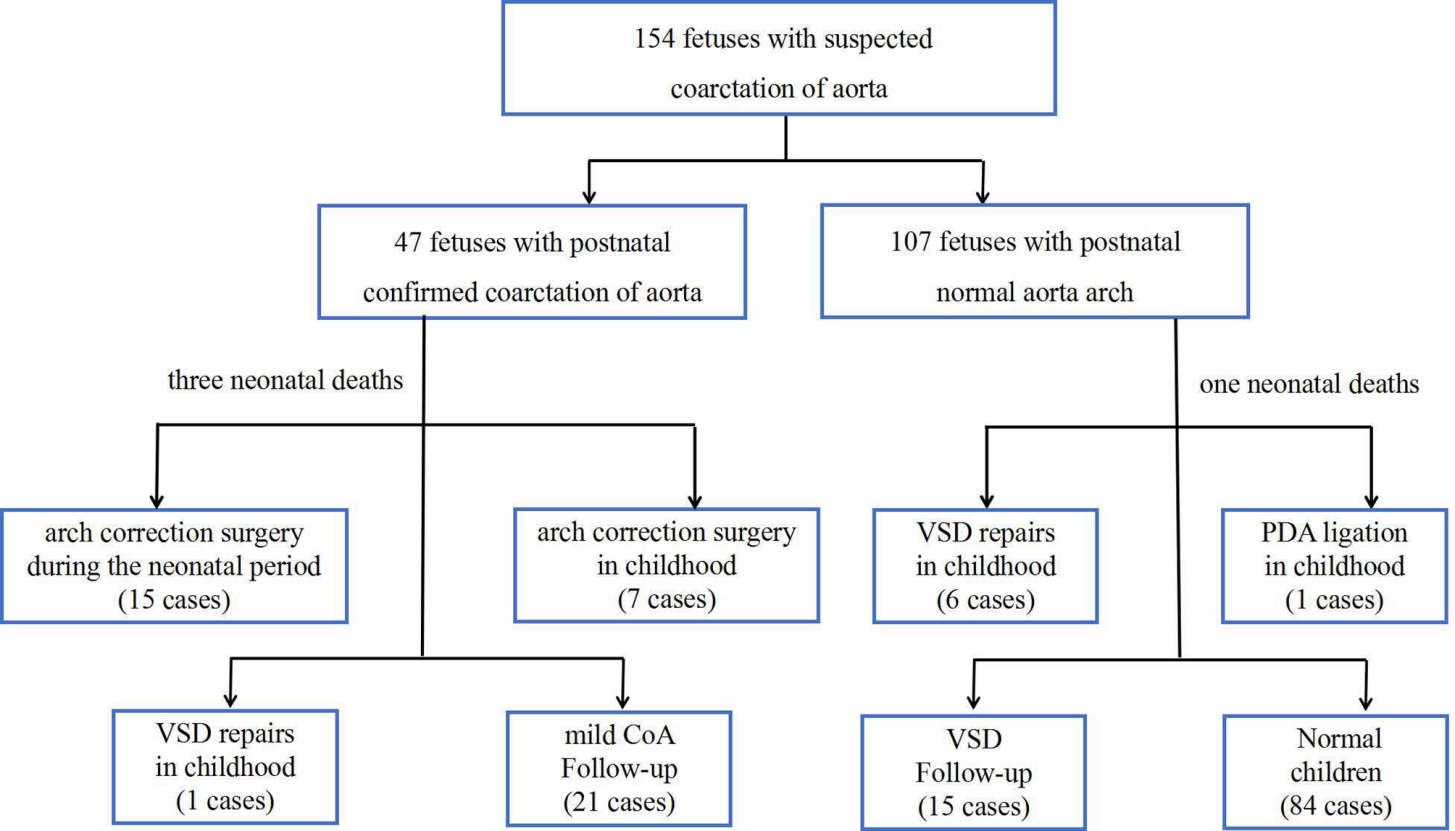
Outcomes of eligible fetal cases

All of the fetuses were born alive, and twenty-nine children underwent cardiac surgeries. Figure 2 shows the outcomes for the entire group. Compared with those with normal arches postnatally, the fetuses with confirmed CoA had a smaller aortic diameter and wider distance from the LSA to LCCA. Confirmed CoA was positively associated with the presence of VSD and PLSVC (Table 1).

**Table 1:** Prenatal characteristics of the fetuses with suspected coarctation of the aorta and echocardiography indexes predicting postnatal coarctation of the aorta

	With confirmed CoA *n* = 47	With normal arch *n* = 107	*P*
Maternal age, years	29 ± 4.3	31 ± 4.6	0.669
Gestational age at diagnosis, weeks	27 ± 2.9	28 ± 4.5	0.079
RVEDD-to-LVEDD ratio	1.27 ± 0.27	1.24 ± 0.21	0.444
RVEDA-to-LVEDA ratio	1.31 ± 0.47	3.14 ± 18.14	0.510
AO-to-PA ratio	0.68 ± 0.13	0.72 ± 0.86	0.145
AV annular diameter	4.36 ± 0.70	4.67 ± 1.03	0.036
Z-score of AV	-0.01 ± 1.15	0.1 ± 0.99	0.573
MV-to-TV ratio	0.79 ± 0.14	0.81 ± 0.10	0.330
Diameter of transverse arch	1.92 ± 0.32	3.06 ± 0.67	0.0001
Z-score of transverse arch	-7.30 ± 1.29	-4.02 ± 1.31	0.0001
Diameter of isthmus	1.43 ± 0.31	2.30 ± 0.58	0.0001
Z-score of isthmus	-8.97 ± 1.45	-5.65 ± 1.60	0.0001
TAO-DAO angle	104.54 ± 11.51	125.29 ± 8.97	0.0001
Diameter of LSA	1.61 ± 0.39	1.74 ± 0.49	0.103
Isthmus-to-LSA ratio	0.92 ± 0.23	1.37 ± 0.35	0.0001
Distance between LCCA and LSA	4.45 ± 1.75	2.74 ± 1.07	0.0001
Distance between RIA and LCCA	1.99 ± 0.79	1.99 ± 0.65	0.980
Ratio of the distances	2.39 ± 0.89	1.41 ± 0.51	0.0001
Presence of VSD	59.6% (28/47)	24.3% (26/107)	0.0001
Presence of PLSVC	23.4% (11/47)	7.5% (8/107)	0.014
Peak aortic velocity	71.39 ± 13.63	68.54 ± 15.54	0.349
Flow direction across IAS			0.447
≥ 3mm	89.4% (42/47)	13.1% (14/107)	
< 3mm	10.6% (5/47)	86.9% (93/107)	
Flow direction across isthmus			0.0001
Antegrade	63.8% (30/47)	90.7% (97/107)	
With retrograde	4.3% (2/47)	3.7% (4/107)	
With delayed passage	31.9% (15/47)	5.6% (6/107)	

RVEDD-to-LVEDD ratio: the ratio of right ventricular end-diastolic dimension to left ventricular end-diastolic dimension; RVEDA-to-LVEDA ratio: the ratio of right ventricular end-diastolic area to left ventricular end-diastolic area; AO-to-PA ratio: the ratio of aortic artery diameter to pulmonary artery diameter; AV: aortic valve; standard score: Z-score; MV-to-TV ratio: ratio of mitral valve annular diameter to tricuspid valve annular diameter; TAO-DAO angle: the angle between the transverse aortic arch and isthmus; LSA: left subclavian artery; isthmus-to-LSA ratio: ratio of isthmus diameter to LSA diameter; LCCA: left common carotid artery; RIA: right innominate artery; VSD: ventricular septal defect; PLSVC: persistent left superior vena cava; R-to-L shunt: right to left shunt

**Fig. 3:**
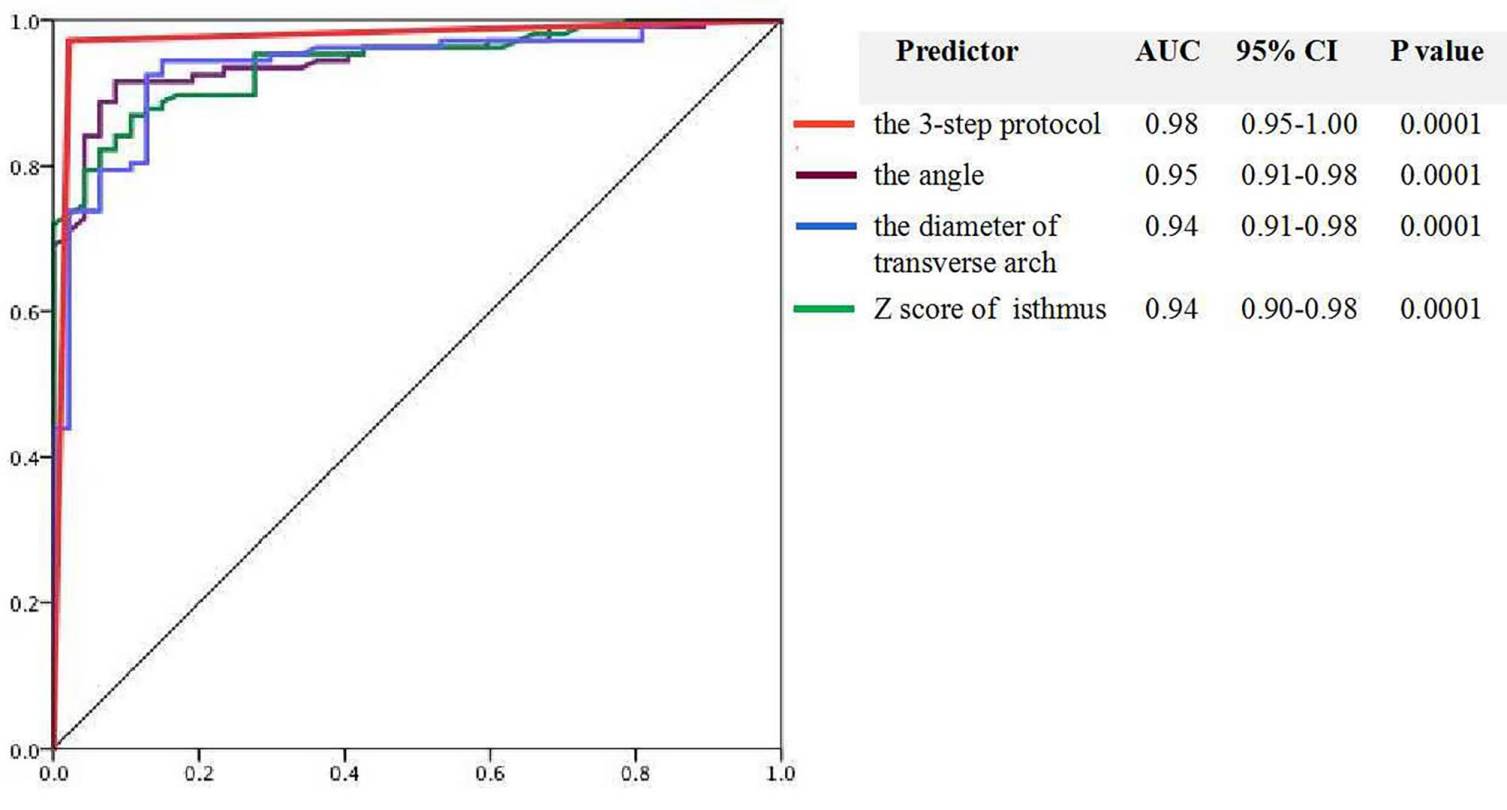
Receiver operating characteristics curve of the 3-step diagnostic protocol and the main three parameters for fetal coarctation of the aorta diagnosis, including the 3-step protocol (red line), the TAO-DAO angle (purple line), the diameter of the transverse arch (blue line), and the Z-score of the isthmus (green line)

The ROC curve determined that the 3-step diagnostic protocol was the strongest indicator of fetal CoA, with area under the curve (AUC) was 0.975 (95% CI 0.95–1.00). The single strong diagnostic indicators were a TAO-DAO angle ≤ 115.75°, a transverse arch diameter ≤ 2.31 mm, and a Z-score of the isthmus ≤ -7.5. The AUC was 0.947 (95% CI 0.91–0.98) for the TAO-DAO angle, 0.942 (95% CI 0.91–0.98) for the transverse arch diameter, and 0.937 (95% CI 0.90–0.98) for the Z-score of the isthmus (Fig. 3). The following indices showed a strong specificity for the diagnosis of fetal CoA: a Z-score of the transverse arch of ≤ -5.29, diameter of the isthmus of ≤ 1.64 mm, distance from the LSA to LCCA of ≥ 3.5 mm, the ratio of isthmus diameter and LSA diameter ≤ 0.89, and ratio of the distances of ≥ 1.81 (Table 2).

**Table 2:** The optimal critical values of echocardiographic indices for fetal coarctation of the aorta diagnosis, determined by receiver operating characteristic (ROC)

Index	Cut-off value	AUC	Sen	Sep
TAO-DAO angle	115.75	0.947	91.6%	91.5%
Diameter of transverse arch	2.31	0.942	86.9%	89.4%
Z-score of transverse arch	-5.29	0.968	86.9%	97.9%
Diameter of isthmus	1.64	0.918	87.9%	83%
Z-score of isthmus	-7.5	0.937	92.5%	87.2%
Ratio of the distances	1.81	0.84	80.9%	85.8%
Distance between LCCA and LSA	3.50	0.823	76.6%	85.8%
Isthmus-to-LSA ratio	0.89	0.758	98.1%	41%

AUC: area under curve; Sen: sensitivity; Sep: specificity; TAO-DAO angle: the angle between the transverse aortic arch and isthmus; standard score: Z-score; LCCA: left common carotid artery; LSA: left subclavian artery

**Fig. 4:**
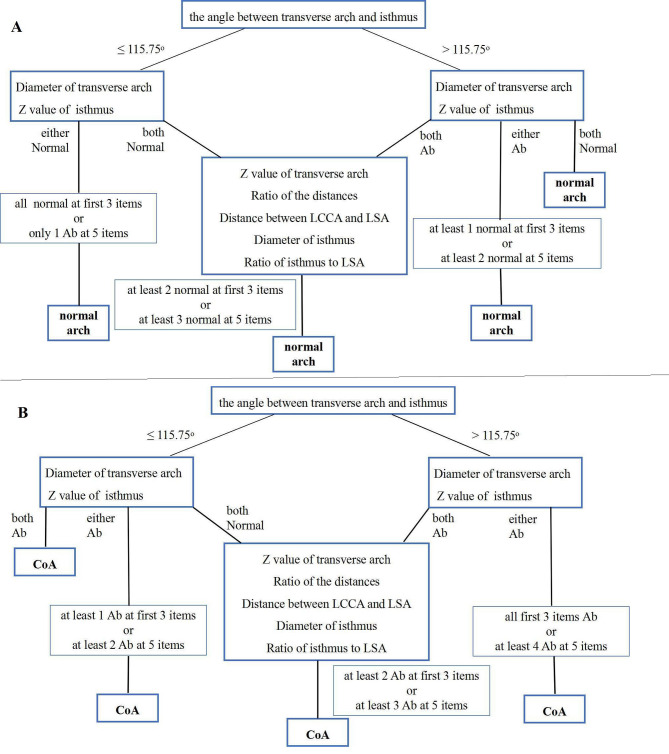
Comprehensive 3-step echocardiographic diagnostic process to identify a normal fetal aortic arch (**A**) and fetal coarctation of the aorta (**B**). LCCA: left common carotid artery; LSA: left subclavian artery

When all of the first three measurements (the TAO-DAO angle, a transverse arch diameter and a Z-score of the isthmus) were abnormal, the diagnosis of fetal CoA was supported in 34 cases (72.3%, 34/47). Conversely, when all of these indices were within the normal range, a postnatal normal arch was predicted in 81 cases (75.7%, 81/107). Subsequently, five additional measurements were utilized to improve specificity in the algorithm. According to their diagnostic performance, the indices were incorporated into a diagnostic flow chart (Fig. 4) that forms the basis of our proposed three-step diagnostic protocol. The kappa test showed that the diagnostic model’s evaluation was consistent with the postnatal outcome of the aortic arch with 97.8% sensitivity and 97.2% specificity (kappa value 0.936, *P* = 0.0001), whereas the accurate diagnosis rate from the two experienced doctors (H.M; L.Z.L.) was 77%.

Four neonatal deaths occurred among the 154 cases due to duodenal atresia (1.3%, 2/154), cerebral anomalies (0.65%, 1/154), and premature birth (0.65%, 1/154). Among the 47 children with postnatally confirmed CoA, 22 children underwent arch corrections, including 15 in the neonatal period and 7 in childhood. Compared with the other 32 children who did not require neonatal intervention, the 15 children with duct-dependent CoA had a smaller transverse arch Z-score (-7.90 ± 1.40 vs. -7.05 ± 1.08; *P* = 0.031) although they had similar other echocardiographic data. Among the 107 children with a normal arch postnatally, six underwent VSD repairs, and one underwent ductus arteriosus ligation. No postoperative deaths occurred. Their echocardiographic and clinical follow-up findings, growth, and development were normal.

## Discussion

It is important to accurately diagnose fetal CoA; however, fetal prediction of postnatal CoA continues to be a challenge [12]. Even several echocardiographic indices are used to diagnose fetal CoA, no single parameter has good diagnostic accuracy [13, 14]. In this retrospective study, we validated that postnatal CoA can be predicted with a high degree of accuracy using a 3-step echocardiographic diagnostic model.

### Step one

Previous studies demonstrated that a tortuous and rigid aortic arch is the most intuitive sign of CoA diagnosis in children. Some researchers have used the TAO-DAO angle to digitally evaluate the arch contour rather than relying on the operator’s subjective evaluation. Drs. Arya [3] and Freeman [15] defined one angle between the ascending and descending aorta (≤ 20.31°) and another angle between the transverse and descending aorta (≥ 96.15°) to detect fetal CoA. These two measurements could not be obtained if the aortic arch was too tortuous or if no standard view of the entire aortic arch was present. So they suggested combining the distance between the LCCA-to-LSA and Z-score of the ascending aorta to decrease the rate of misdiagnosis.

Coarctation occurs mostly at the isthmus, so a change in the angle due to a tortuous or rigid lumen is most likely to occur at this point, which may explain why the TAO-DAO angle between the transverse arch and isthmus in our study was found to be the best index to judge the shape of the arch, as it had the largest AUC (0.95) and the best sensitivity and specificity (both 92%). Difficulty in clearly displaying the entire arch contour in one view may lead to incorrect measurements, which may explain why we found that some fetuses with confirmed CoA had a normal TAO-DAO angle or that a fetus with a postnatal normal arch had an abnormal TAO-DAO angle. We found the TAO-DAO angle as the main index in the diagnostic protocol but still utilized the subsequent two steps to minimize errors in diagnosis.

### Step two

The diameter of the transverse arch was measured, and the Z-score of the isthmus was calculated. In agreement with published data [13, 16], we found that the presence of a hypoplastic aortic arch had a good diagnostic performance in detecting CoA. Since the diameter of transverse arch and isthmus must be different at respective gestational age, the Z-scores of the aorta were calculated. Considering that a 0.5 mm difference in the isthmus diameter may result in a Z-score of − 2 instead of − 1, we prefer to propose that the Z-scores, instead of the absolute data, should be used to increase the diagnostic sensitivity to 92.5%, while concurrently using the transverse arch diameter to improve the specificity to 89.4%. The two indices used together would balance any false negative and false positive rates. Gómez-Montes [7] proposed the cut-off value of the Z-score of the isthmus to be ≤ -2 when measured in 3VT view, which is larger than our data. In our study, the measurement of the isthmus had to be taken at the insertion of the PDA in the sagittal and coronary views or proximal to the orifice of the LSA in the long axis view, and the cut-off value was ≤ -7.5. We hold that a strict protocol is more suitable for clear display and standard measurements, facilitating diagnostic accuracy.

The first three indices used in steps 1 and 2, representing the arch contour and dimension, could differentiate the cases of true narrowing from those of suspected CoA most of the time. When all of these indices were abnormal, the diagnosis of fetal CoA was supported, and conversely, when all of these indices were within the normal range, a postnatal normal arch was predicted. Among the 47 fetuses with true CoA, the accurate diagnosis rate was 72.3% (34/47), when all the first three measurements were abnormal. Conversely, when all these indices were within the normal range, a postnatal normal arch was predicted in 81 cases among 107 false-positive cases (75.7%).

### Step Three

When the two-step measurements were completed, the diagnosis of CoA was still undetermined with 24.3% false-negative fetus and 27.7% false-positive fetus. So the following five indices should be added to improve the diagnostic accuracy further.

1. The distance from the LCCA to the LSA always increased in patients with CoA, which is theorized to be secondary to a stretch in the contour of the transverse arch [17]. Therefore, we measured the distances and calculated the ratio. The distance from the LCCA to the LSA and ratio of the distances had a high specificity of 86%, which could reduce the false-positive rate and provide an indirect evaluation of the arch contour.
2. The aortic arch was further assessed using the Z-score of the transverse arch and the diameter of the isthmus [18]. The two indices compensated for the contradictory judgment in step 2. A reduced Z-score of the transverse arch would strongly support the diagnosis of fetal CoA. If isthmus stenosis is associated with a smaller transverse arch’s Z-score, the duct-dependent CoA that requires intervention in the neonatal period should be considered.
3. Prior studies have indicated that the diameter of the isthmus should be smaller than that of the LSA in fetal CoA [19]. Therefore, the isthmus-to-LSA ratio is considered an important clue to the diagnosis of fetal CoA, which was confirmed in our study. However, the isthmus-to-LSA ratio had a lowest specificity (41%). Therefore, the isthmus-to-LSA ratio may suggest CoA, but it is not specific enough to independently diagnose CoA. The diagnosis must be made in conjunction with more specific indices.

Dr. Dodge-Khatami [17] first defined the carotid-subclavian artery index as the ratio of the isthmus diameter to the distance between the LCCA and LSA, and proved that the parameters had high sensitivity and specificity for detecting fetal CoA [20]. In our study, the isthmus diameter and the distance between the LCCA and LSA were also assessed in this algorithm.

We found that the arterial duct often appears as a tortuous aneurysm in CoA, especially in the third trimester. The tortuous arterial duct is often mistaken for the aortic arch, resulting in both a missed diagnosis and misdiagnosis. Therefore, a key point of prenatal echocardiography is to clearly show the isthmus where the LSA originates or the arterial duct inserts.

A close relationship between postnatal arch intervention and a higher peak ascending aorta Doppler velocity (OR, 2.51, per 20 cm/s; *P* = 0.001) was found in a previous study [21]. One explanation could be that the pulsed wave Doppler with the best alignment at the ascending aorta just above the AV is easier to record, whereas Doppler tracing of the transverse aortic arch or isthmus is difficult due to the tortuous and stenotic lumen and the angle-dependence. In our study, abnormal flow direction across the isthmus was found in the fetuses with true CoA, however, the index was not put into the algorithm due to low diagnostic sensitivity. In future research, the peak Doppler velocity across the aorta should be recorded to explore the diagnostic sensitivity and realizability.

In addition, Dr Fruitman [22] also established a score to predict CoA, including gestational age, Z-score of RV mid-cavitary dimension, and Z-score of isthmal diameter, which presented a good diagnostic performance with 90% sensitivity and 83% specificity. However, gestational age, RV dimension and its Z-score were not confirmed as indicators of fetal CoA in our study. One possible explanation could be that the measurement of RV dimension is non-standard, since there are at least two different fetal four-chamber views (the apical 4-chamber view and the subcostal 4-chamber view).

The proposed algorithm in our study is better than the other scoring systems [17–20, 22] due to the standardized measurements. Recently, some researchers have reported that three-dimensional printing and speckle-tracking peak global longitudinal strain might be helpful in detecting fetal congenital heart diseases [23, 24]. These novel tools could be verified in future research.

## Limitation

The proposed algorithm has not been tested in more patients. In the future, the algorithm based on our retrospective study requires to be validated by a prospective multicenter registry of suspected CoA fetuses. Furthermore, the main purpose of this proposed algorithm was not to detect the fetuses with PGE-dependent CoA requiring neonatal coarctation correction. In this retrospective study, 29 children met the diagnostic criteria and did not undergo neonatal arch correction. In further studies, a larger number of cases should be included to specify the algorithm to predict the severity of CoA and the timing of intervention.

## Conclusion

The 3-step diagnostic protocol starts with a measurement of the aortic TAO-DAO angle, followed by measurements of the transverse arch and isthmus, and finally incorporates additional indices. The algorithm is useful for the detection of aortic coarctation in fetuses with a high degree of accuracy.

## Data Availability

The datasets used and/or analyzed during the current study are available from the corresponding author on reasonable request.

## Abbreviations

CoA: Coarctation of the aorta
PDA: Patent ductus arteriosus
CHD: Congenital heart defect
CT: Computed tomography
RV: Right ventricular
RVEDD: Right ventricular end diastolic dimension
RVEDA: Right ventricular end diastolic area
LV: Left ventricular
LVEDD: Left ventricular end diastolic dimension
LVEDA: Left ventricular end diastolic area
MV: Mitral valve
TV: Tricuspid valve
AV: Aortic valve
PV: Pulmonary valve
AO: Aortic artery
PA: Pulmonary artery
3VT: Three vessels and trachea view
LSA: Left subclavian artery
LCCA: Left common carotid artery
RIA: Right innominate artery
Z-score: Standard score
ROC: Receiver operating characteristic
AUC: Area under the curve
CI: Confidence interval
VSD: Ventricular septal defects
PLSVC: Persistent left superior venae cavae
